# 1,5-Dimethyl-2-phenyl-4-{[(*E*)-3,4,5-trimethoxybenzylidene]amino}-1*H*-pyrazol-3(2*H*)-one

**DOI:** 10.1107/S160053681002934X

**Published:** 2010-07-31

**Authors:** Shan-Bin Liu, Cai-Feng Bi, Yu-Hua Fan, Xia Zhang, Dong-Mei Zhang

**Affiliations:** a Key Laboratory of Marine Chemistry Theory and Technology, Ministry of Education, College of Chemistry and Chemical Engineering, Ocean University of China, Qingdao, Shandong 266100, People’s Republic of China

## Abstract

In the title compound, C_21_H_23_N_3_O_4_, the pyrazole ring forms dihedral angles of 21.58 (8) and 66.64 (7)° with the benzene and phenyl rings, respectively. The crystal structure is stabilized by weak inter­molecular C—H⋯O hydrogen bonds.

## Related literature

For general background to Schiff base compounds, see: Atwood & Harvey (2001 ▶); Che & Huang (2003 ▶). For the applications of metal–Schiff base complexes, see: Drozdzak *et al.* (2005 ▶); Adsule *et al.* (2006 ▶); Yuan *et al.* (2007 ▶). For a related structure, see: Sun *et al.* (2007 ▶).
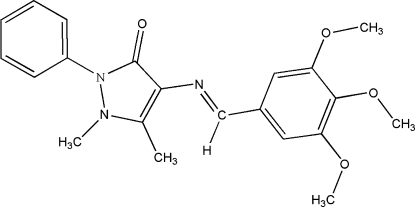


## Experimental

### 

#### Crystal data

C_21_H_23_N_3_O_4_
*M* *_r_* = 381.42
Monoclinic, 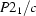

*a* = 12.3644 (12) Å
*b* = 14.0075 (16) Å
*c* = 11.2682 (11) Å
β = 96.4680 (1)°
*V* = 1939.2 (3) Å^3^
*Z* = 4
Mo *K*α radiation
μ = 0.09 mm^−1^
*T* = 298 K
0.40 × 0.17 × 0.13 mm

#### Data collection

Siemens SMART CCD diffractometer
Absorption correction: multi-scan (*SADABS*; Sheldrick, 1996 ▶) *T*_min_ = 0.964, *T*_max_ = 0.988
10089 measured reflections
3422 independent reflections
2084 reflections with *I* > 2σ(*I*)
*R*_int_ = 0.038

#### Refinement

*R*[*F* ^2^ > 2σ(*F* ^2^)] = 0.042
*wR*(*F* ^2^) = 0.098
*S* = 0.99
3422 reflections
258 parameters
H-atom parameters constrained
Δρ_max_ = 0.15 e Å^−3^
Δρ_min_ = −0.18 e Å^−3^

### 

Data collection: *SMART* (Siemens, 1996 ▶); cell refinement: *SAINT* (Siemens, 1996 ▶); data reduction: *SAINT*; program(s) used to solve structure: *SHELXS97* (Sheldrick, 2008 ▶); program(s) used to refine structure: *SHELXL97* (Sheldrick, 2008 ▶); molecular graphics: *SHELXTL* (Sheldrick, 2008 ▶) and *PLATON* (Spek, 2009 ▶); software used to prepare material for publication: *SHELXTL*.

## Supplementary Material

Crystal structure: contains datablocks global, I. DOI: 10.1107/S160053681002934X/lh5091sup1.cif

Structure factors: contains datablocks I. DOI: 10.1107/S160053681002934X/lh5091Isup2.hkl

Additional supplementary materials:  crystallographic information; 3D view; checkCIF report

## Figures and Tables

**Table 1:** Hydrogen-bond geometry (Å, °)

*D*—H⋯*A*	*D*—H	H⋯*A*	*D*⋯*A*	*D*—H⋯*A*
C5—H5*C*⋯O1^i^	0.96	2.31	3.211 (2)	155
C9—H9⋯O4^ii^	0.93	2.56	3.346 (3)	142

Symmetry codes: (i) 
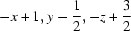
; (ii) 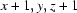.

**Fig. 1.**
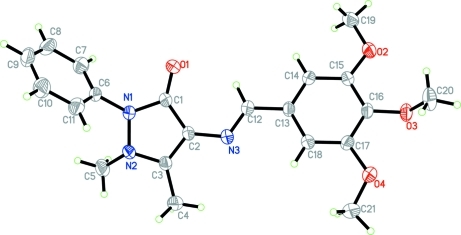
The molecular structure of the title compound, showing 30% probability displacement ellipsoids and the atom-numbering scheme.

**Fig. 2.**
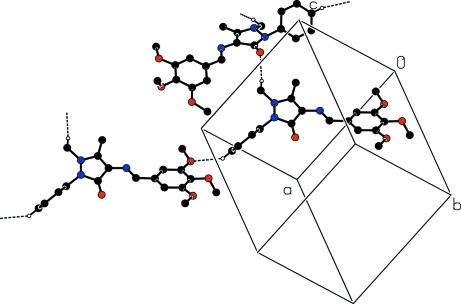
Part of the crystal structure showing weak C—H···O hydrogen bonds as dashed lines. Only H atoms involved in hydrogen bonds are shown.

## supplementary crystallographic information

### Comment

Schiff bases are among the most fundamental chelating systems in coordination chemistry (Atwood *et al.*, 2001; Che *et al.*, 2003). The metal complexes based on this type ligands have expanded enormously areas of catalytic activities (Drozdzak *et al.*, 2005), molecular magnetism (Yuan *et al.*, 2007) and biological activities, such as antitumor activities (Adsule *et al.*, 2006). The examples given above clearly demonstrate that Schiff base ligands are of special interest in the field of chemistry. Herein, we present the synthesis and crystal structure of the title compound.

The molecular structure of the title compound is shown in Fig. 1. The bond lengths and angles can be compared to those in a related structure (Sun *et al.*, 2007).  The dihedral angles between the pyrazole ring and the benzene and phenyl rings are 21.58 (8)° and 66.64 (7)°, respectively. The crystal structure is stabilized by by weak intermolecular C—H···O hydrogen bonds.

### Experimental

4-aminoantpyrine (10 mmol, 2.032 g) was added with stirring to anhydrous ethanol (30 ml) and an anhydrous ethanol solution (10 ml) of 3,4,5-trimethoxybenzaldehyde (10 mmol, 1.962 g) was slowly added. The reaction mixture was stirred at 353 K for 5 h, whereupon a yellow solid separated out. The precipitate formed was filtered off, washed several times with anhydrous ethanol and dried under vacuum. Yellow block-shaped crystals were obtained from an anhydrous ethanol solution of the title compound after 2 days by slow evaporation at room temperature.

### Refinement

All H-atoms were positioned geometrically and refined using a riding model, with C—H = 0.93 - 0.96 Å *U*_iso_(H) = 1.2*U*_eq_(C) or 1.5*U*_eq_(C_methyl_).

### Crystal data

**Table:**

C_21_H_23_N_3_O_4_	*F*(000) = 808
*M**_r_* = 381.42	*D*_x_ = 1.306 Mg m^−^^3^
Monoclinic, *P*2_1_/*c*	Mo *K*α radiation, λ = 0.71073 Å
Hall symbol: -P 2ybc	Cell parameters from 2128 reflections
*a* = 12.3644 (12) Å	θ = 2.2–25.3°
*b* = 14.0075 (16) Å	µ = 0.09 mm^−^^1^
*c* = 11.2682 (11) Å	*T* = 298 K
β = 96.4680 (1)°	Block, yellow
*V* = 1939.2 (3) Å^3^	0.40 × 0.17 × 0.13 mm
*Z* = 4	

### Data collection

**Table:**

Siemens SMART CCD diffractometer	3422 independent reflections
Radiation source: fine-focus sealed tube	2084 reflections with *I* > 2σ(*I*)
graphite	*R*_int_ = 0.038
φ and ω scans	θ_max_ = 25.0°, θ_min_ = 1.7°
Absorption correction: multi-scan (*SADABS*; Sheldrick, 1996)	*h* = −14→14
*T*_min_ = 0.964, *T*_max_ = 0.988	*k* = −16→12
10089 measured reflections	*l* = −13→13

### Refinement

**Table:**

Refinement on *F*^2^	Primary atom site location: structure-invariant direct methods
Least-squares matrix: full	Secondary atom site location: difference Fourier map
*R*[*F*^2^ > 2σ(*F*^2^)] = 0.042	Hydrogen site location: inferred from neighbouring sites
*wR*(*F*^2^) = 0.098	H-atom parameters constrained
*S* = 0.99	*w* = 1/[σ^2^(*F*_o_^2^) + (0.039*P*)^2^] where *P* = (*F*_o_^2^ + 2*F*_c_^2^)/3
3422 reflections	(Δ/σ)_max_ < 0.001
258 parameters	Δρ_max_ = 0.15 e Å^−^^3^
0 restraints	Δρ_min_ = −0.18 e Å^−^^3^

### Special details

**Table:**

Geometry. All e.s.d.'s (except the e.s.d. in the dihedral angle between two l.s. planes) are estimated using the full covariance matrix. The cell e.s.d.'s are taken into account individually in the estimation of e.s.d.'s in distances, angles and torsion angles; correlations between e.s.d.'s in cell parameters are only used when they are defined by crystal symmetry. An approximate (isotropic) treatment of cell e.s.d.'s is used for estimating e.s.d.'s involving l.s. planes.
Refinement. Refinement of *F*^2^ against ALL reflections. The weighted *R*-factor *wR* and goodness of fit *S* are based on *F*^2^, conventional *R*-factors *R* are based on *F*, with *F* set to zero for negative *F*^2^. The threshold expression of *F*^2^ > σ(*F*^2^) is used only for calculating *R*-factors(gt) *etc*. and is not relevant to the choice of reflections for refinement. *R*-factors based on *F*^2^ are statistically about twice as large as those based on *F*, and *R*- factors based on ALL data will be even larger.

### Fractional atomic coordinates and isotropic or equivalent isotropic displacement parameters (Å^2^)

**Table:**

	*x*	*y*	*z*	*U*_iso_*/*U*_eq_	
N1	0.58862 (12)	0.17160 (11)	0.75617 (14)	0.0483 (4)	
N2	0.55362 (12)	0.07674 (11)	0.73659 (14)	0.0488 (4)	
N3	0.38480 (12)	0.18932 (11)	0.49987 (13)	0.0456 (4)	
O1	0.56035 (11)	0.31372 (9)	0.65254 (13)	0.0616 (4)	
O2	0.14379 (11)	0.51801 (9)	0.24428 (12)	0.0597 (4)	
O3	0.06093 (10)	0.38623 (9)	0.08868 (12)	0.0555 (4)	
O4	0.12137 (12)	0.20441 (10)	0.10776 (12)	0.0641 (4)	
C1	0.53561 (15)	0.22988 (14)	0.66664 (17)	0.0451 (5)	
C2	0.45678 (14)	0.16819 (13)	0.60050 (16)	0.0415 (5)	
C3	0.46934 (14)	0.07841 (13)	0.64717 (17)	0.0425 (5)	
C4	0.40708 (16)	−0.00978 (13)	0.61267 (19)	0.0568 (6)	
H4A	0.4568	−0.0613	0.6042	0.085*	
H4B	0.3628	0.0004	0.5382	0.085*	
H4C	0.3614	−0.0255	0.6733	0.085*	
C5	0.55506 (17)	0.01888 (15)	0.8440 (2)	0.0682 (7)	
H5A	0.5077	0.0468	0.8963	0.102*	
H5B	0.6278	0.0162	0.8838	0.102*	
H5C	0.5306	−0.0445	0.8227	0.102*	
C6	0.70012 (16)	0.18510 (13)	0.80311 (17)	0.0460 (5)	
C7	0.72641 (19)	0.24765 (15)	0.89492 (19)	0.0645 (6)	
H7	0.6722	0.2807	0.9286	0.077*	
C8	0.8353 (2)	0.26125 (18)	0.9374 (2)	0.0788 (8)	
H8	0.8544	0.3047	0.9985	0.095*	
C9	0.9143 (2)	0.2109 (2)	0.8894 (3)	0.0798 (8)	
H9	0.9871	0.2200	0.9182	0.096*	
C10	0.88698 (18)	0.14758 (18)	0.7999 (2)	0.0765 (7)	
H10	0.9411	0.1129	0.7683	0.092*	
C11	0.78040 (17)	0.13441 (15)	0.7557 (2)	0.0613 (6)	
H11	0.7622	0.0913	0.6939	0.074*	
C12	0.36413 (15)	0.27582 (14)	0.47241 (17)	0.0478 (5)	
H12	0.3986	0.3235	0.5201	0.057*	
C13	0.28837 (15)	0.30404 (13)	0.36894 (16)	0.0429 (5)	
C14	0.25703 (15)	0.39875 (13)	0.35636 (17)	0.0463 (5)	
H14	0.2868	0.4437	0.4114	0.056*	
C15	0.18193 (15)	0.42704 (13)	0.26269 (17)	0.0439 (5)	
C16	0.13902 (15)	0.36017 (14)	0.17957 (17)	0.0446 (5)	
C17	0.17022 (15)	0.26509 (14)	0.19268 (16)	0.0460 (5)	
C18	0.24460 (15)	0.23685 (14)	0.28662 (16)	0.0461 (5)	
H18	0.2653	0.1731	0.2947	0.055*	
C19	0.17650 (18)	0.58758 (14)	0.33332 (19)	0.0658 (6)	
H19A	0.1578	0.5659	0.4092	0.099*	
H19B	0.1400	0.6468	0.3129	0.099*	
H19C	0.2538	0.5970	0.3379	0.099*	
C20	0.10337 (18)	0.41981 (17)	−0.01563 (19)	0.0735 (7)	
H20A	0.1388	0.4801	0.0009	0.110*	
H20B	0.0450	0.4275	−0.0787	0.110*	
H20C	0.1549	0.3745	−0.0396	0.110*	
C21	0.12836 (19)	0.10573 (14)	0.1296 (2)	0.0703 (7)	
H21A	0.2030	0.0859	0.1341	0.105*	
H21B	0.0864	0.0722	0.0660	0.105*	
H21C	0.1005	0.0917	0.2038	0.105*	

### Atomic displacement parameters (Å^2^)

**Table:**

	*U*^11^	*U*^22^	*U*^33^	*U*^12^	*U*^13^	*U*^23^
N1	0.0450 (10)	0.0437 (10)	0.0528 (11)	−0.0016 (8)	−0.0099 (8)	0.0052 (8)
N2	0.0480 (10)	0.0402 (10)	0.0556 (11)	−0.0017 (8)	−0.0053 (8)	0.0121 (9)
N3	0.0477 (10)	0.0446 (10)	0.0427 (10)	0.0030 (7)	−0.0027 (8)	0.0051 (8)
O1	0.0657 (10)	0.0377 (9)	0.0756 (11)	−0.0008 (7)	−0.0176 (8)	0.0059 (7)
O2	0.0717 (10)	0.0454 (9)	0.0582 (9)	0.0105 (7)	−0.0101 (8)	0.0030 (7)
O3	0.0515 (9)	0.0663 (10)	0.0453 (9)	0.0047 (7)	−0.0093 (7)	0.0096 (7)
O4	0.0802 (11)	0.0523 (10)	0.0538 (9)	−0.0018 (7)	−0.0188 (8)	−0.0043 (8)
C1	0.0445 (12)	0.0400 (12)	0.0493 (13)	0.0061 (9)	−0.0022 (9)	0.0020 (10)
C2	0.0419 (11)	0.0415 (12)	0.0397 (12)	0.0043 (9)	−0.0018 (9)	0.0029 (9)
C3	0.0392 (11)	0.0427 (12)	0.0450 (12)	0.0038 (9)	0.0019 (9)	0.0027 (10)
C4	0.0553 (13)	0.0479 (13)	0.0663 (15)	−0.0039 (10)	0.0024 (11)	0.0001 (11)
C5	0.0662 (15)	0.0656 (15)	0.0695 (16)	−0.0030 (12)	−0.0064 (12)	0.0279 (13)
C6	0.0470 (12)	0.0438 (12)	0.0441 (12)	−0.0020 (10)	−0.0088 (10)	0.0079 (10)
C7	0.0706 (16)	0.0645 (15)	0.0557 (14)	0.0010 (12)	−0.0050 (12)	−0.0033 (12)
C8	0.089 (2)	0.0772 (18)	0.0617 (16)	−0.0245 (16)	−0.0266 (15)	−0.0002 (14)
C9	0.0563 (16)	0.085 (2)	0.091 (2)	−0.0145 (14)	−0.0253 (15)	0.0310 (17)
C10	0.0463 (15)	0.0813 (18)	0.101 (2)	0.0010 (12)	0.0043 (14)	0.0130 (16)
C11	0.0518 (14)	0.0638 (15)	0.0668 (16)	0.0008 (11)	−0.0005 (12)	−0.0037 (12)
C12	0.0491 (12)	0.0481 (13)	0.0437 (12)	0.0007 (10)	−0.0051 (9)	0.0011 (10)
C13	0.0425 (11)	0.0478 (12)	0.0367 (11)	0.0008 (9)	−0.0027 (9)	0.0051 (10)
C14	0.0513 (12)	0.0453 (12)	0.0403 (12)	−0.0034 (9)	−0.0038 (9)	0.0006 (9)
C15	0.0455 (12)	0.0415 (11)	0.0437 (12)	0.0031 (9)	0.0001 (9)	0.0061 (10)
C16	0.0407 (11)	0.0524 (13)	0.0387 (12)	0.0022 (9)	−0.0039 (9)	0.0072 (10)
C17	0.0500 (12)	0.0494 (13)	0.0372 (12)	−0.0040 (10)	−0.0012 (10)	−0.0016 (10)
C18	0.0515 (12)	0.0425 (12)	0.0429 (12)	0.0027 (9)	−0.0010 (10)	0.0035 (10)
C19	0.0833 (17)	0.0464 (13)	0.0663 (15)	0.0053 (12)	0.0020 (13)	−0.0007 (12)
C20	0.0790 (17)	0.0973 (19)	0.0432 (14)	0.0217 (14)	0.0019 (12)	0.0140 (13)
C21	0.0875 (18)	0.0523 (15)	0.0675 (16)	−0.0072 (12)	−0.0063 (13)	−0.0076 (12)

### Geometric parameters (Å, °)

**Table:**

N1—C1	1.402 (2)	C8—C9	1.364 (3)
N1—N2	1.407 (2)	C8—H8	0.9300
N1—C6	1.432 (2)	C9—C10	1.357 (3)
N2—C3	1.366 (2)	C9—H9	0.9300
N2—C5	1.455 (2)	C10—C11	1.368 (3)
N3—C12	1.269 (2)	C10—H10	0.9300
N3—C2	1.392 (2)	C11—H11	0.9300
O1—C1	1.228 (2)	C12—C13	1.465 (3)
O2—C15	1.367 (2)	C12—H12	0.9300
O2—C19	1.424 (2)	C13—C14	1.385 (2)
O3—C16	1.375 (2)	C13—C18	1.388 (2)
O3—C20	1.420 (2)	C14—C15	1.383 (3)
O4—C17	1.368 (2)	C14—H14	0.9300
O4—C21	1.405 (2)	C15—C16	1.387 (3)
C1—C2	1.445 (3)	C16—C17	1.390 (3)
C2—C3	1.365 (2)	C17—C18	1.380 (2)
C3—C4	1.484 (2)	C18—H18	0.9300
C4—H4A	0.9600	C19—H19A	0.9600
C4—H4B	0.9600	C19—H19B	0.9600
C4—H4C	0.9600	C19—H19C	0.9600
C5—H5A	0.9600	C20—H20A	0.9600
C5—H5B	0.9600	C20—H20B	0.9600
C5—H5C	0.9600	C20—H20C	0.9600
C6—C7	1.367 (3)	C21—H21A	0.9600
C6—C11	1.376 (3)	C21—H21B	0.9600
C7—C8	1.391 (3)	C21—H21C	0.9600
C7—H7	0.9300		
			
C1—N1—N2	109.06 (15)	C9—C10—H10	119.7
C1—N1—C6	122.78 (15)	C11—C10—H10	119.7
N2—N1—C6	116.67 (14)	C10—C11—C6	119.8 (2)
C3—N2—N1	107.16 (14)	C10—C11—H11	120.1
C3—N2—C5	124.06 (15)	C6—C11—H11	120.1
N1—N2—C5	114.90 (16)	N3—C12—C13	122.99 (18)
C12—N3—C2	119.61 (16)	N3—C12—H12	118.5
C15—O2—C19	117.73 (15)	C13—C12—H12	118.5
C16—O3—C20	114.23 (15)	C14—C13—C18	119.85 (18)
C17—O4—C21	118.35 (16)	C14—C13—C12	119.09 (18)
O1—C1—N1	123.15 (18)	C18—C13—C12	121.01 (17)
O1—C1—C2	131.93 (18)	C15—C14—C13	120.58 (18)
N1—C1—C2	104.86 (16)	C15—C14—H14	119.7
C3—C2—N3	122.96 (17)	C13—C14—H14	119.7
C3—C2—C1	108.14 (16)	O2—C15—C14	125.01 (18)
N3—C2—C1	128.69 (17)	O2—C15—C16	115.34 (17)
C2—C3—N2	110.13 (16)	C14—C15—C16	119.65 (17)
C2—C3—C4	129.25 (18)	O3—C16—C15	120.43 (17)
N2—C3—C4	120.61 (17)	O3—C16—C17	119.79 (18)
C3—C4—H4A	109.5	C15—C16—C17	119.65 (18)
C3—C4—H4B	109.5	O4—C17—C18	124.20 (18)
H4A—C4—H4B	109.5	O4—C17—C16	115.18 (17)
C3—C4—H4C	109.5	C18—C17—C16	120.62 (18)
H4A—C4—H4C	109.5	C17—C18—C13	119.64 (18)
H4B—C4—H4C	109.5	C17—C18—H18	120.2
N2—C5—H5A	109.5	C13—C18—H18	120.2
N2—C5—H5B	109.5	O2—C19—H19A	109.5
H5A—C5—H5B	109.5	O2—C19—H19B	109.5
N2—C5—H5C	109.5	H19A—C19—H19B	109.5
H5A—C5—H5C	109.5	O2—C19—H19C	109.5
H5B—C5—H5C	109.5	H19A—C19—H19C	109.5
C7—C6—C11	120.3 (2)	H19B—C19—H19C	109.5
C7—C6—N1	120.07 (19)	O3—C20—H20A	109.5
C11—C6—N1	119.66 (18)	O3—C20—H20B	109.5
C6—C7—C8	119.1 (2)	H20A—C20—H20B	109.5
C6—C7—H7	120.4	O3—C20—H20C	109.5
C8—C7—H7	120.4	H20A—C20—H20C	109.5
C9—C8—C7	120.1 (2)	H20B—C20—H20C	109.5
C9—C8—H8	120.0	O4—C21—H21A	109.5
C7—C8—H8	120.0	O4—C21—H21B	109.5
C10—C9—C8	120.2 (2)	H21A—C21—H21B	109.5
C10—C9—H9	119.9	O4—C21—H21C	109.5
C8—C9—H9	119.9	H21A—C21—H21C	109.5
C9—C10—C11	120.5 (2)	H21B—C21—H21C	109.5
			
C1—N1—N2—C3	−8.40 (19)	C8—C9—C10—C11	0.9 (4)
C6—N1—N2—C3	−152.94 (16)	C9—C10—C11—C6	−0.6 (4)
C1—N1—N2—C5	−150.54 (16)	C7—C6—C11—C10	−0.8 (3)
C6—N1—N2—C5	64.9 (2)	N1—C6—C11—C10	179.61 (18)
N2—N1—C1—O1	−170.55 (17)	C2—N3—C12—C13	−179.20 (16)
C6—N1—C1—O1	−28.6 (3)	N3—C12—C13—C14	169.61 (17)
N2—N1—C1—C2	6.95 (19)	N3—C12—C13—C18	−7.8 (3)
C6—N1—C1—C2	148.88 (17)	C18—C13—C14—C15	0.3 (3)
C12—N3—C2—C3	168.73 (17)	C12—C13—C14—C15	−177.09 (17)
C12—N3—C2—C1	−17.2 (3)	C19—O2—C15—C14	−5.6 (3)
O1—C1—C2—C3	174.1 (2)	C19—O2—C15—C16	173.74 (17)
N1—C1—C2—C3	−3.0 (2)	C13—C14—C15—O2	178.25 (17)
O1—C1—C2—N3	−0.6 (3)	C13—C14—C15—C16	−1.1 (3)
N1—C1—C2—N3	−177.84 (17)	C20—O3—C16—C15	88.4 (2)
N3—C2—C3—N2	173.05 (16)	C20—O3—C16—C17	−95.6 (2)
C1—C2—C3—N2	−2.1 (2)	O2—C15—C16—O3	−2.1 (3)
N3—C2—C3—C4	−6.2 (3)	C14—C15—C16—O3	177.30 (16)
C1—C2—C3—C4	178.61 (18)	O2—C15—C16—C17	−178.04 (16)
N1—N2—C3—C2	6.4 (2)	C14—C15—C16—C17	1.3 (3)
C5—N2—C3—C2	144.21 (18)	C21—O4—C17—C18	14.1 (3)
N1—N2—C3—C4	−174.22 (15)	C21—O4—C17—C16	−165.26 (17)
C5—N2—C3—C4	−36.4 (3)	O3—C16—C17—O4	2.5 (3)
C1—N1—C6—C7	86.2 (2)	C15—C16—C17—O4	178.47 (17)
N2—N1—C6—C7	−134.48 (18)	O3—C16—C17—C18	−176.90 (16)
C1—N1—C6—C11	−94.1 (2)	C15—C16—C17—C18	−0.9 (3)
N2—N1—C6—C11	45.2 (2)	O4—C17—C18—C13	−179.14 (17)
C11—C6—C7—C8	1.8 (3)	C16—C17—C18—C13	0.2 (3)
N1—C6—C7—C8	−178.59 (18)	C14—C13—C18—C17	0.1 (3)
C6—C7—C8—C9	−1.5 (3)	C12—C13—C18—C17	177.48 (17)
C7—C8—C9—C10	0.2 (4)		

### Hydrogen-bond geometry (Å, °)

**Table:**

*D*—H···*A*	*D*—H	H···*A*	*D*···*A*	*D*—H···*A*
C5—H5C···O1^i^	0.96	2.31	3.211 (2)	155
C9—H9···O4^ii^	0.93	2.56	3.346 (3)	142

Symmetry codes: (i) −*x*+1, *y*−1/2, −*z*+3/2; (ii) *x*+1, *y*, *z*+1.
